# Tumor Lysis Syndrome Following Thoracotomy Under Cardiopulmonary Bypass in a Case of Hepatocellular Carcinoma With Right Atrial and Inferior Vena Cava Tumor Thrombus

**DOI:** 10.7759/cureus.20311

**Published:** 2021-12-09

**Authors:** Juey-Ming Shih

**Affiliations:** 1 Critical Care Medicine, Cathay General Hospital, Taipei, TWN; 2 Nutrition and Health Science, College of Nutrition, Taipei Medical University, Taipei, TWN

**Keywords:** tumor lysis syndrome, open heart surgery, disseminated intravascular coagulopathy, continuous renal replacement therapy (crrt), cardiopulmonary bypass, inferior vena cava tumor thrombus, right atrium tumor thrombus, hepatocellular carcinoma

## Abstract

Tumor lysis syndrome (TLS) occurring after surgical resection of right atrium (RA) and inferior vena cava (IVC) tumor thrombus is a very rare but insidious condition. We report a case of hepatocellular carcinoma who developed TLS after uneventful excision of RA+IVC tumor thrombus under median sternotomy and cardiopulmonary bypass (CPB). Although the procedure was not expected to arouse massive tumor cell necrosis, post-operative course was complicated by metabolic acidosis, hypocalcemia, and progressive hyperkalemia indicative of TLS. Unfortunately, laboratory diagnosis of TLS was delayed under conditions of continuous renal replacement therapy (CRRT) for peri-operative acute renal failure. Despite all efforts, the patient died 36 hours after surgery due to lethal arrhythmia and disseminated infarction of the kidneys, spleen, and liver.

## Introduction

Hepatocellular carcinoma (HCC) represents the majority of all primary liver cancers, the second leading cause of cancer-related deaths in the world [1]. About 2.4-6.3% of patients with HCC develop tumor thrombus involving the inferior vena cava (IVC) and right atrium (RA), a rare manifestation that can lead to sudden death from pulmonary embolism or right heart failure [2]. Traditionally, the median survival for untreated patients of HCC with RA tumor thrombus is only one to five months after diagnosis [3]. With advancements in surgical techniques, aggressive resection of both hepatic and RA tumor thrombus under cardiopulmonary bypass (CPB) can be safely performed prolonging median survival up to 30.8 months after curative resection [3]. Thus, aggressive intervention is becoming a feasible option for salvaging these patients.

Tumor lysis syndrome (TLS) is a life-threatening condition caused by massive destruction of tumor cells more frequently seen after chemotherapy for highly proliferative hematologic malignancies such as acute lymphoblastic leukemia or high-grade lymphoma rather than solid tumors like HCC [4]. Following tumor cell lysis, metabolites are released into the systemic circulation resulting in severe electrolyte abnormalities, acute renal failure, metabolic acidosis, seizures, multi-organ failure, and even death [5]. Although the overall incidence of TLS can be as high as 26.4% among B-cell acute lymphoblastic leukemia [4], TLS stemming from solid tumors, in contrast, is quite rare and usually presented as sporadic cases [6]. Nonetheless, the literature has increasingly reported cases among cancers of the lung, breast, gastrointestinal tract, liver, urogenital and gynecological organs, and even the skin [6]. This progressive increase may be primarily attributed to recent advancements in the treatment of these solid tumors. Despite its low incidence, TLS stemming from solid tumors have a relatively high mortality rate at around 35%, a figure much higher than the 1.9% mortality rate seen in their hematologic counterparts [7]. Similar to hematologic malignancies, solid tumors usually develop TLS after cancer-targeted cytotoxic interventions such as radiation, chemotherapy, radiofrequency, or chemoembolization [6]. While spontaneous TLS can occur intra-operatively for solid tumors, TLS arising after open-heart surgery has never been documented [8]. We report a case of HCC with IVC+RA tumor thrombus who received successful tumor thrombus excision via median thoracotomy and under cardiopulmonary bypass. Post-operatively, TLS developed with eventual progression into intractable hyperkalemia and fatal cardiac dysrhythmia. Awareness of possible TLS after open-heart tumor thrombectomy may assist cardiac surgeons and intensivists in the timely management of this oncological emergency.

## Case presentation

A 79-year-old female patient with underlying conditions of hypertension, coronary artery disease, and hepatitis C presented with progressive bilateral legs edema up to the lower abdomen over several weeks. She initially visited our cardiology outpatient department where echocardiography was performed. During the study, a large space-occupying lesion was noted with near-total obstruction of the RA. She was immediately referred to our emergency department for management. Subsequent contrast-enhanced computed tomography (CECT) revealed a large hypervascular tumor in segment 8 of the liver with tumor extension into the hepatic vein, IVC, RA, and right ventricle (RV). She then disclosed that she had been diagnosed with solitary HCC eight months earlier but had refused treatment. The patient was then admitted to our surgical intensive care unit (ICU) for further management.

After admission, low molecular weight heparin (LMWH) was prescribed. In addition, an empirical antibiotic was provided due to suspected urinary tract infection (UTI) and right lower lobe (RLL) pneumonia. Two days after admission, oliguria was noted with poor response to hydration and furosemide use. Continuous renal replacement therapy (CRRT) was initiated due to anuria, progressive dyspnea, and intermittent hypotension. Repeat CECT showed stationary HCC with tumor thrombus and near-total obstruction of the suprarenal IVC and RA. There was no evidence of tumor emboli or infarction of the lungs, kidneys, or liver. Under the impression of low cardiac output syndrome due to cardiac inflow obstruction, resection of the RA+IVC tumor thrombus was arranged and uneventfully completed via median sternotomy and under CPB. Briefly, the CPB circuitry was established by cannulating the ascending aorta, the superior vena cava via the right atrium, and the inferior vena cava via the right common femoral vein. With the heart arrested, an extended right atriotomy was performed to expose the high-integrity tumor mass. Two CPB pump suctions were positioned inside the atriotomy wound to remove blood and possible emboli during the tumor excision. Arterial filters were used in the CPB circuit to prevent microemboli from reentering the circulation. Concomitant hepatectomy was not performed with plans for trans-arterial embolization (TAE) and chemotherapy after the palliative procedure. A few hours after surgery, progressive metabolic acidosis and hemodynamic instability complicated her clinical course but was initially manageable under CRRT and low-dose inotropic use. However, conscious change and severe hypotension (requiring high-dose inotropic drugs) erupted about 12 hours after the procedure. Mediastinal thoracotomy drains totaled 350 mL/12 hours. Brain and abdominal CECT follow-up showed no evidence of stroke nor intra-abdominal tumor bleeding. Subsequent serologic studies showed hypoxemia, metabolic acidosis, and hepatic failure but her vitals were sustainable under our treatment. Hypocalcemia was witnessed but potassium levels remained normal under CRRT so TLS was overlooked. Hyperkalemia ultimately developed late in her clinical course and was irresponsive even to aggressive CRRT dosage adjustments. A review of her CECT identified disseminated infarction of the kidneys, spleen, and liver. TLS was finally diagnosed but all efforts to correct hyperkalemia and metabolic acidosis were in vain. About 36 hours after the open-heart procedure, bradycardia with wide QRS complexes and hyper T waves developed progressing rapidly to ventricular fibrillation (VF) and pulseless electrical activity (PEA). Despite all efforts at resuscitation, the patient expired.

## Discussion

HCC with IVC and RA tumor thrombosis

IVC and RA tumor thrombosis occurs in only about 0.67-4.1% of patients with HCC [9], but case numbers seem to be increasing as a result of improved survival and progressive advances in imaging techniques [10]. Contrary to general expectations, the disease process is often insidious and without primary cardiac symptoms. Instead, patients usually present with more non-specific symptoms such as cachexia, edema, abdominal distention or pain, and dyspnea secondary to pulmonary embolism or Budd-Chiari syndrome [11]. As a result, clinical diagnosis may sometimes be delayed until the IVC and RA masses are relatively large making intervention more difficult and the treatment course more complicated.

Owing to technical limitations in the past, surgical resection of the RA tumor thrombus had resulted in post-operative survival of only one to nine months (mean: six months) [9]. However, more recent studies have shown improved results, especially among Child-Pugh class A patients following both surgical and non-surgical interventions. With improvements in surgical techniques, it is now possible to perform curative resection by simultaneously resecting both the intrahepatic primary tumor and the IVC and/or RA tumor thrombus under CPB to achieve a median survival of up to 30.8 months [3]. In addition, advances in combined radiation (RT) and trans-arterial chemoembolization (TACE) therapy have also shown median survival rates of up to 21 months [12].

Our patient initially presented with progressive bilateral leg and truncal edema without cardiac symptoms mainly as a result of significant IVC tumor obstruction. A few days after admission, acute renal failure developed followed by respiratory distress and hypotension. Resection of RA+IVC tumor thrombus was performed under CPB in hopes of quickly improving her hemodynamics. Laparotomy with curative hepatic resection was not attempted, but plans for TAE and chemotherapy were made to treat her hepatoma once her conditions stabilized. Although the thoracotomy procedure was uneventful, TLS, unfortunately, erupted after the procedure with rapid progression into disseminated intra-vascular coagulation (DIC) and multi-organ failure (MOF).

Tumor lysis syndrome

Tumor lysis syndrome (TLS) is a life-threatening condition caused by massive destruction of tumor cells that can occur spontaneously but more frequently as a complication of cancer therapy for rapidly proliferating and chemo-sensitive hematologic malignancies [7]. As a result of cell destruction, metabolites are released into the systemic circulation leading to fatal metabolic abnormalities including hyperkalemia, hyperphosphatemia, hyperuricemia, and hypocalcemia [7]. TLS stemming from solid tumors, such as HCC, is rare primarily because of their resistance to cytotoxic therapies [5]. Despite its low incidence, TLS from solid tumors is associated with a much higher mortality rate (around 35%) compared to that of TLS among hematologic malignancies (around 1.9%) [7]. Although HCC represents a mere 8% of all solid tumors with documented TLS [13], the mortality rate of TLS from HCC can be as high as 50% owing to poor recognition and delayed diagnosis [5]. Similar to hematologic malignancies, solid tumors can develop TLS spontaneously or after cancer-targeted interventions such as radiation, chemotherapy, radiofrequency, or chemoembolization [6]. However, TLS following open-heart procedure under CPB has yet to be reported.

The diagnosis of TLS requires one clinical and two laboratory abnormalities according to the Cairo-Bishop criteria [14]. Laboratory TLS includes hyperuricemia, hyperkalemia, hyperphosphatemia, and hypocalcemia (exceeding respective limits of the norm) noted within three days prior to and seven days after initiation of cytotoxic therapy. A 25% change from baseline serum levels is also accepted as a significant deviation from the norm. Clinical TLS is present when laboratory TLS is accompanied by one or more of the following conditions: acute renal injury (increased creatinine level >1.5x norm), seizures, cardiac dysrhythmia, or sudden death [14]. Interestingly, patients may present with renal dysfunction prior to acquiring TLS and this precondition usually yields grave prognosis [4].

The most important aspect of treatment is timely identification of the patients at risk for TLS so that prophylactic measures can be initiated early. Tumors that are large and bulky, widely metastatic, or more susceptible to cytoreductive therapy are at higher risk for TLS. Tumors with high-serum lactate dehydrogenase (LDH) levels, which may indicate rapid cell turnover, are also more likely to develop TLS. More importantly, pre-existing renal disease not only increases the risk of developing TLS but also can worsen TLS-associated complications due to the kidney’s inability to cope with the metabolic effects of tumor lysis [4].

The management of clinical TLS is generally supportive, involving strict analyte monitoring, aggressive hydration to keep adequate urine output, and early use of hypouricemic agents such as allopurinol or rasburicase to prevent fulminant renal injury. Once electrolyte derangements occur, rapid correction of hyperkalemia and hypocalcemia is needed along with vigilant cardiac monitoring. Hemodialysis is frequently required in the event of acute renal failure which is associated with high mortality [4]. In general, most clinical manifestations can be managed when recognized early. Delayed recognition and untimely management of TLS complications can be life-threatening even when intensive hemodialysis is provided, as evident in this patient.

TLS, DIC, and MOF

Although acute kidney injury has traditionally been cited as the most common cause of death in patients with TLS from solid tumors, growing evidence suggests that disseminated microemboli from the debris of lysed tumor cells may be involved in promoting micro-obstruction of multi-organ capillary beds including kidneys, brain, and the lungs ultimately leading to widespread tissue ischemia, necrosis, and multi-organ failure [7]. The presence of DIC among cancer patients is not new. DIC has been estimated to be in 15-20% of patients with acute leukemia and 7-15% of patients with solid metastatic tumors [15]. Furthermore, several studies support the hypothesis that DIC and TLS can coexist, with one seemingly providing a nidus for the other, and contribute to a rapid synergistic decline in patients’ hemodynamic status. Takeuchi et al. reported a case of malignant melanoma with spontaneous TLS and rapid deterioration within 40 hours of admission [16]. Autopsy of this patient revealed disseminated microthrombi in the portal system, but more importantly, massive necrosis of normal hepatocytes was noted alongside melanoma cell death within the liver [16]. In another study, Ito et al. reported a case of recurrent endometrial cancer presenting with TLS about 19 hours after initiation of docetaxel and carboplatin therapy [17]. In addition to hyperuricemia, hyperkalemia, and hypocalcemia, the patient showed symptoms of chest pain, tachypnea, and oliguria. Echocardiography revealed right heart strain but computed tomography indicated no evidence of pulmonary embolism. Although hyperkalemia and metabolic acidosis were managed successfully, right-sided heart failure ultimately led to her death 54 hours after chemotherapy. Autopsy confirmed disseminated microemboli in what was otherwise a normal lung [17].

TLS and DIC are both processes orchestrated by inflammatory mediators. During TLS, rapid cell lysis causes the release of pro-inflammatory cytokines such as interleukin-6 (IL-6), IL-8, IL-1, and tumor necrosis factor-α (TNF-α) leading to nitric oxide-mediated vasodilatation and systemic inflammation [7]. This cytokine storm can also activate DIC by inducing tissue factor (TF) overexpression on monocytes, endothelial cells, and even some tumor cells [18]. TF, the major trigger of coagulation in DIC, both promotes and propagates excessive thrombin generation. In addition to TF upregulation, DIC is characterized by platelet activation, defective anticoagulation mechanisms, insufficient or exaggerated fibrin degradation, and concomitant activation of the inflammatory process. Ultimately, it is the diffuse microvascular obstruction in conjunction with hemodynamic and metabolic derangements of the inflammatory process that leads to multi-organ dysfunction and death [18]. Previous studies have demonstrated the presence of pro-inflammatory cytokines even following single courses of chemotherapy for acute myeloid leukemia [19]. Incidentally, cytokine release has also been identified in the serum of patients undergoing CPB. This cytokine release is primarily triggered by ischemic-reperfusion injury, and the elevations in myocardial IL-6, IL-8, and TNF-α have all been shown to be in direct correlation with the duration of cardiac ischemia [20]. This case suffers from several risk factors for TLS including a large and bulky tumor mass, and pre-operative acute renal failure requiring CRRT. Under these conditions, it is possible that the systemic inflammatory response from open-heart surgery, and more importantly CPB use, may have been sufficient to induce hypercytokinemia, which is the pivotal pathophysiologic trigger for TLS and DIC. Although spontaneous TLS could have occurred in our patient, it seems more reasonable to speculate that open-heart surgery under CPB may have triggered a cytokine storm leading to TLS, DIC, and MOF several hours after the procedure.

**Figure 1:**
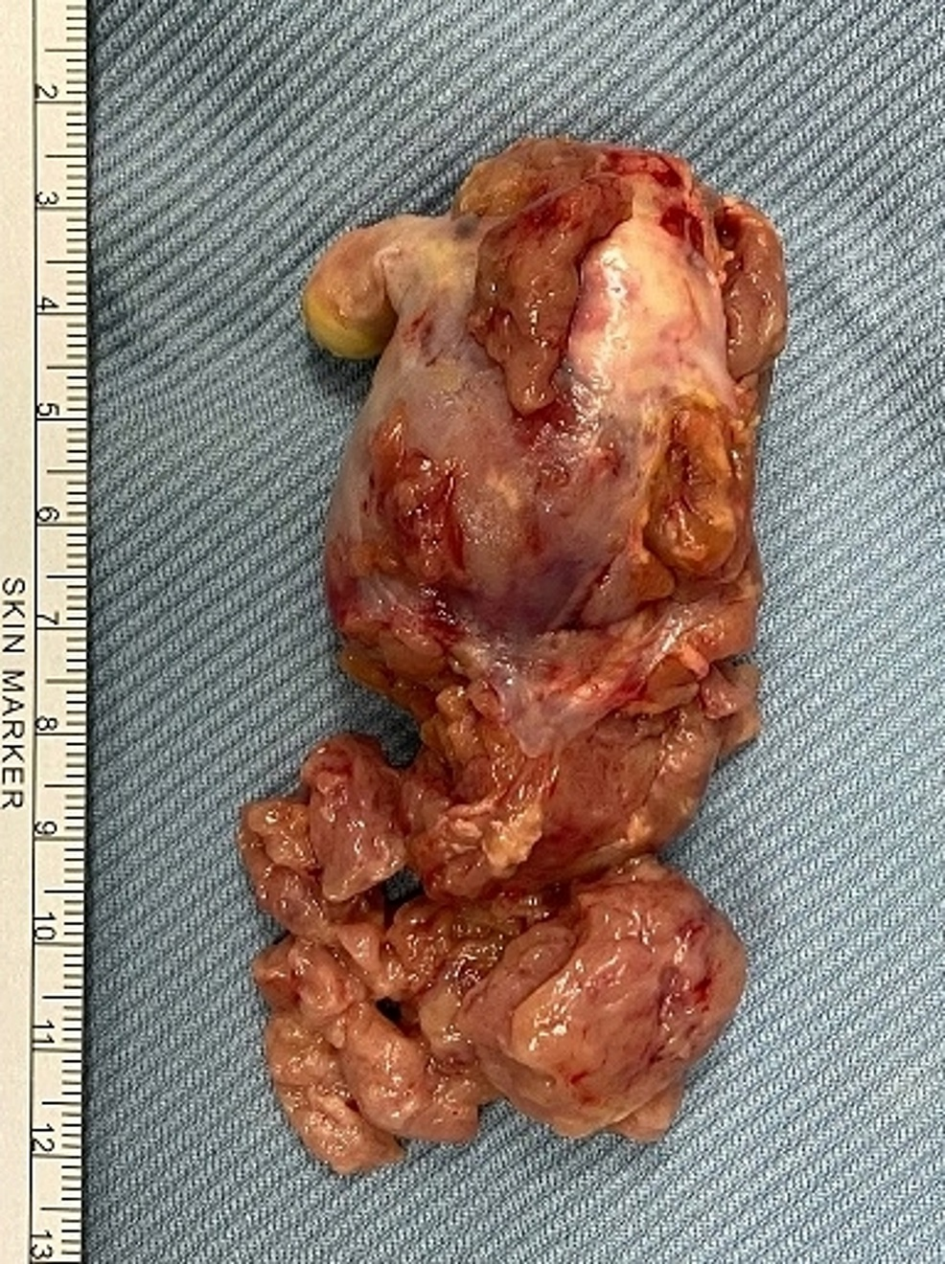
HCC tumor thrombus Hepatocellular carcinoma (HCC) tumor thrombus measuring 8.5x5.5x3.8 cm excised en bloc from the right atrium and inferior vena cava.

In this patient, anuria developed one day prior to surgical intervention and was managed with CRRT. Although pre-existent renal dysfunction is a critical risk factor, TLS was not expected because the patient did not undergo any cytotoxic treatment for her HCC. The RA and IVC tumor thrombus was removed en bloc without difficulty via median sternotomy and under CPB (Figure 1). Her primary hepatic tumor mass was left untouched. Although spontaneous intra-operative TLS have been previously reported in literature, neither laboratory nor clinical TLS was witnessed during the open-heart procedure [8]. Shortly after surgery, hypocalcemia developed but without hyperkalemia marking the possible onset of laboratory TLS. Because CRRT was initiated immediately after surgery, progressive hyperkalemia and metabolic acidosis may have been concealed to delay the timely diagnosis of TLS.

**Figure 2:**
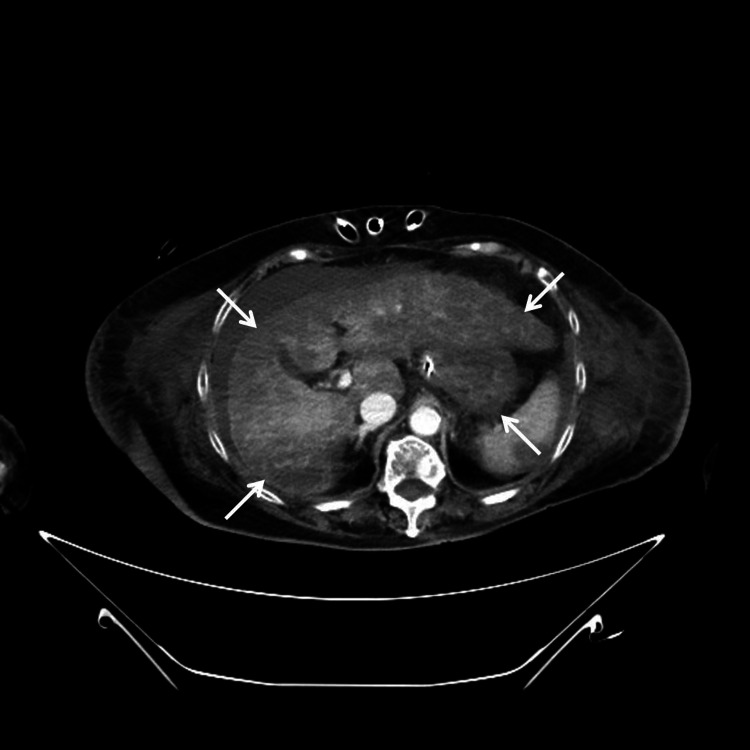
Contrast-enhanced CT, transverse section of the liver Contrast-enhanced computed tomography (CT) showed diffuse areas of non-enhancement in the liver (white arrows).

**Figure 3:**
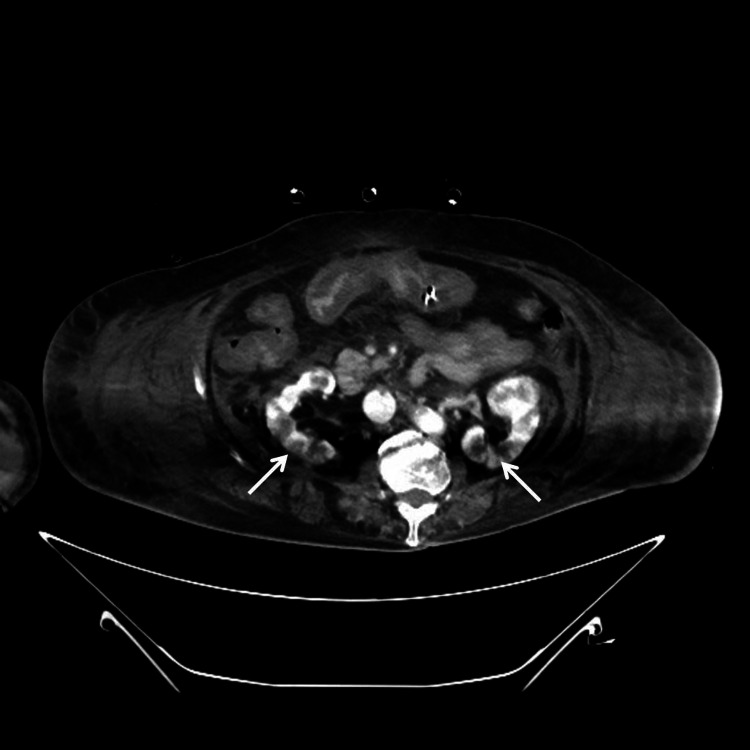
Contrast-enhanced CT, transverse section of the kidneys Contrast-enhanced computed tomography (CT) showed scattered regions of non-enhancement over bilateral kidneys (white arrows).

**Figure 4:**
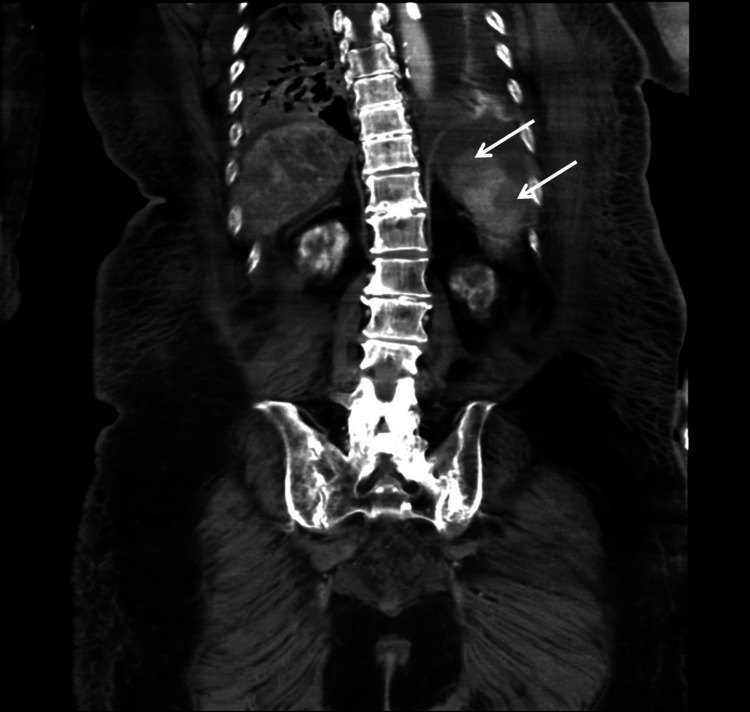
Contrast-enhanced CT, coronal section of the spleen Contrast-enhanced computed tomography (CT) showed regions of non-enhancement in the spleen (white arrows).

About 12 hours after surgery, deterioration of consciousness and hemodynamics was noted in the absence of hemorrhagic shock. Interestingly, computed tomography study at the time showed evidence of disseminated infarction of the liver, kidneys, and spleen, as well as leukoaraiosis in the brain suspicious of ischemia (Figures 2-4). TLS with DIC should have been considered but was overlooked. Under high-dose CRRT, hyperkalemia and fulminant metabolic acidosis finally developed about 30 hours after surgery. TLS with MOF was confirmed but all efforts at salvaging the patient were in vain. Repeated episodes of fatal arrhythmia erupted, and the patient expired about 36 hours after the procedure.

## Conclusions

TLS occurring after surgical management for HCC with IVC+RA tumor thrombus is a rare and insidious condition that can easily go unnoticed. This is especially true in the modern intensive care unit where early and aggressive correction of electrolyte abnormalities and acid-base derangements using CRRT is often the standard of care. Under such conditions, laboratory characteristics of TLS may not provide an effective and timely means of diagnosis. With an improved understanding of the inflammatory nature of TLS, it may be possible in the near future to develop better methods of detecting TLS or even identifying those at risk for DIC and MOF. Although the cause of TLS in this case remains speculative and requires further research, we hope that this case report can raise the awareness of TLS among cardiovascular surgeons treating patients with RA+IVC tumor thrombus under CPB.
